# Editorial: Vaginal microecological disorder and gynecological diseases

**DOI:** 10.3389/fcimb.2023.1292815

**Published:** 2023-10-18

**Authors:** Tingtao Chen, Ruonan Wu, Lei Cheng, Qinping Liao, Zhangran Chen

**Affiliations:** ^1^ National Engineering Research Center for Bioengineering Drugs and the Technologies, Institute of Translational Medicine, Nanchang University, Nanchang, China; ^2^ Biological Science Division, Pacific Northwest National Laboratory, Richland, WA, United States; ^3^ Key Laboratory of Development and Application of Rural Renewable Energy, Biogas Institute of Ministry of Agriculture and Rural Affairs, Chengdu, China; ^4^ Department of Obstetrics and Gynecology, Beijing Tsinghua Changgung Hospital, School of Clinical Medicine, Tsinghua University, Beijing, China; ^5^ Shenzhen Wedge Microbiology Research Co.Ltd, Shenzhen, China

**Keywords:** vaginal microbiota, uterine microbiota, human papillomavirus, vulvovaginal candidiasis, bacterial vaginitis

Researchers have been increasingly interested in investigating microbial communities in the lower and upper reproductive tract and their impact on female reproductive health. A healthy vaginal ecosystem is dominated by *Lactobacillus* spp., which can be infected by various pathogens, including human papillomavirus (HPV) and human immunodeficiency virus, and is susceptible to dysbiosis of its microbiota. Pathogenic overgrowth results in the development of diseases, including aerobic vaginitis, bacterial vaginosis(BV), cytolytic vaginosis, trichomonas vaginitis, vulvovaginal candidiasis (VVC), urinary tract infections, sexually transmitted infections (STIs), and gynecological oncology. Determining the associations between the vaginal microbiota and these diseases can provide valuable insights for detecting diagnosing, and interventing complex female illnesses.

This Research Topic comprises fifteen papers, consisting of three subtopics: vaginal inflammation, HPV, and reproductive health.

The first subtopic on vaginal inflammation, comprises one review, two BV-related research articles, and two VVC-related articles. Gao et al. observed the top five potential common pathogens of vaginal infection, with *Haemophilus influenzae* having the highest prevalence, followed by *Streptococcus pyogenes*, *Candida albicans*, *Escherichia coli*, and *Staphylococcus aureus*. Zhou et al. examined the vaginal microbiota of patients with BV before and after antibiotic treatment, compared with healthy controls and identified *Lactobacillus iners* as a potential predictive indicator of clinical outcomes in patients with BV. Shen et al. showed that a postbiotic gel alleviated BV symptoms by increasing *Lactobacillus* spp., and reducing the presence of potential vaginal pathogens. Sun et al. summarized the changes in vaginal microbiota during VVC infection and highlighted the potential use of *Lactobacillus* spp. as probiotics for VVC treatment. Considering the high costs and potential side effects associated with antifungal agents for VVC treatment, Lu et al. found that X33 antimicrobial oligopeptide (X33 AMOP) effectively inhibited the virulence of *C.albicans* by reducing phospholipase activity and disrupting mycelium formation. Notably, while *Lactobacillus* spp. has shown promise in preventing and treating vaginal inflammation, clinical data regarding its efficacy remain limited and require further exploration.

Four papers discuss the subtopic of HPV and its relationship with cervical cancer. A et al. discovered a higher prevalence of vaginal infections and cervical STIs in the HPV-positive group than in the HPV-negative group. To explore the impact of probiotics on HPV persistence and clearance, Zeng et al. compared 90 patients with HPV and 45 healthy individuals and found that probiotics, as an interferon adjuvant therapy, effectively enhanced virus clearance in some patients. Patients with HPV clearance had significantly lower alpha diversity, accompanied by a decreased abundance of *Fusobacterium*, *Bacteroides*, *Neisseria* and *Helicobacter*, than those in the HPV-persistent group. Zhu et al. observed negative correlation between interleukin-2 (IL-2) levels and the risk of cervical intraepithelial neoplasia in Chinese women, regardless of high-risk HPV infection. Furthermore, Li et al. found that vaginal microecological abnormalities might contribute to a higher false-positive diagnosis rate of atypical squamous cells of undetermined significance, which is diagnosed as precancerous lesions. These findings highlight the significant impact of vaginal microbiota on female health, emphasizing the importance of understanding and maintaining a balanced microbiome.

Six papers were included in the last subtopic of reproductive health. Chao et al. collected uterine lavage samples via hysteroscopy from women with endometrial hyperplasia (EH) or endometrial cancer (EC) and found an increased relative abundance of two plastic-degrading bacteria, *Bacillus pseudofirmus*, and *Stenotrophomonas rhizophila*, in the endometrial lavage microbiota of women with EC/EH. Liang et al. analyzed the uterine cavity, cervix, and vagina samples of 134 patients with infertility and revealed that endometrial microbiota composed of *Staphylococcus*, *Gardnerella*, *Atobor*, *Streptococcus, Peptostreptococcus*, *Chlamydia*, *Fusobacterium* and *Acinetobacter* are related to CE and EP. Wang et al. discovered that vaginal bacteria, including *Ensifer*, *Devosia*, *Bosea*, *Cellomonas*, *Helicobacter*, and *Sphingopyxis*, as well as specific endometrial microbiota, including *Candidatus Symbiobacter*, *Odoribacter*, *Blautia*, *Nocardioides*, and *Ileibacterium*, exhibited predictive value for embryo arrest. Xie et al. found that vaginal microbiota transfer to newborns could help restore the disturbed microbiome caused by cesarean section delivery, resulting in a microbial composition similar to that of infants born through vaginal delivery. Dong et al. focused on recent advancements in understanding the interactions between microbiota and the cervical mucosal barrier. Furthermore, they found a significant impact of host-microbiota interactions on STIs outcomes. For instance, *Chlamydia trachomatis* infection, can cause tubal inflammation, fibrosis, and even obstruction, which have adverse effects on pregnancy. Tian et al. found that oral antibiotics can induce gut dysbiosis in DBA2/J mice, contributing to the development of *Chlamydia-*induced hydrosalpinx in the upper genital tract.

We reviewed recent research efforts that could further guide the discovery of the underlying mechanisms of microbial-mediated vaginal diseases and provide readers with valuable insights into the prediction, prevention, and treatment of gynecological diseases through reproductive tract microorganisms. In the future, more rigorous and clinically focused research is necessary to explore the specific mechanisms by which microorganisms contribute to the occurrence and development of female diseases. A deeper understanding of these mechanisms will provide crucial information for the diagnosis, treatment strategies, and development and optimization of drugs, probiotics, postbiotics, and vaginal microbiota transplantation.

## Author contributions

TC: Writing – original draft, Writing – review & editing. RW: Writing – review & editing. LC: Writing – review & editing. QL: Writing – review & editing. ZC: Resources, Writing – original draft, Writing – review & editing.

